# Rectal prolapse associated with anorexia nervosa: a case report and review of the literature

**DOI:** 10.1186/2050-2974-1-39

**Published:** 2013-10-10

**Authors:** Nadine Mitchell, Mark L Norris

**Affiliations:** 1 Department of Pediatrics, Children’s Hospital of Eastern Ontario, University of Ottawa, Ottawa, ON Canada; 2 Division of Adolescent Medicine, Children’s Hospital of Eastern Ontario, University of Ottawa, Ottawa, ON Canada; 3 Children’s Hospital of Eastern Ontario Research Institute, Ottawa, ON, Canada

**Keywords:** Anorexia nervosa, Bulimia nervosa, Constipation, Rectal prolapse

## Abstract

Anorexia nervosa is one of a few mental health diagnoses that affects every organ system. Patients with AN often present with multiple secondary effects of starvation at the time of first assessment, including gastrointestinal (GI) complaints. In extreme cases, severe GI complications such as rectal prolapse may be encountered as a consequence of the illness although formal studies investigating the frequency of such occurrences are lacking. We present the case of a 16 year old female previously diagnosed with anorexia nervosa that developed a rectal prolapse as a consequence of her disease as well as a detailed literature review investigating the frequency and prevalence of such occurrences in this population.

## Background

Anorexia nervosa (AN) is a potentially devastating disease that carries high rates of psychological and medical morbidity. Recognized as having the highest mortality rate among psychiatric diagnoses, AN is characterized by a pronounced state of starvation coupled with an intense fear of becoming fat or gaining weight [1]. Despite being under-weight, patients are uniformly distorted in their thoughts and feelings surrounding body image [1]. The prevalence of AN is approximately 1% in industrialized society with an overwhelming female predominance [2]. Although multiple etiological underpinnings have been implicated in disease origins, prevailing wisdom points to a multi-factorial cause implicating biological, psychological, social and environmental influences in vulnerable individuals [1].

Anorexia nervosa is one of a few mental health diagnoses that affects every organ system. If left untreated, severe medical morbidity and complications become the rule not the exception. Patients with AN often present with multiple secondary effects of starvation at the time of first assessment, including gastrointestinal (GI) complaints [3,4]. In almost all cases, gut symptoms improve as patients are renourished and re-establish healthy weights. In extreme cases, severe GI complications such as rectal prolapse may be encountered as a consequence of the illness although formal study investigating the relative frequency of such occurrences is non-existent. The objective of the following article is to present a case-report on a young woman diagnosed with AN that developed rectal prolapse as a consequence of her disease and to conduct a formal review of all published literature investigating the frequency and prevalence of such occurrences in this population cohort.

## Method

A comprehensive literature review using Pubmed, Ovid and Medline databases examining articles relating to AN and rectal prolapse published between January 1st, 1969 and December 31st, 2011 was completed. A total of five medical subject headings (MeSH) were used in different groupings. These included AN, eating disorder (ED), bulimia nervosa (BN), rectal prolapse, and constipation. Relevant abstracts published in the English language were reviewed in depth along with corresponding reference sets.

## Results

A total of 83 abstracts were reviewed, of which 16 matched the objective of the study and were examined in-depth. Articles which focused on the finding of rectal prolapse and BN were more common than those with AN, although only 12 patients across all ED diagnoses were identified. In 1997 Malik et al. published a case series on seven patients with BN and hypothesized that chronically high intra-abdominal pressure associated with vomiting and straining, prolonged gut transit time, and constipation contributed to the rectal prolapse observed [5]. Guerdjikova and colleagues published a case history of another BN patient that experienced rectal prolapse although in this case, rectal purging (repeated finger evacuation of feces in the rectum) was suspected to be the primary contributor [6]. In 2001 Dreznik et al. published a case series describing three young women with AN and rectal prolapse [7]. The three women described were all diagnosed with AN at a young age, suffered from chronic constipation requiring routine laxatives or enemas, and experienced rectal prolapse four to seven years into the course of their respective eating disorder. Ravneet and Paradiso also presented a brief report describing a patient with AN that developed rectal prolapse following long-term laxative abuse [8].

### Case report

A 16 year old female diagnosed three years prior with anorexia nervosa binge-purge subtype presented to the emergency department (ER) of a tertiary care hospital with a chief complaint of constipation. The patient’s eating disorder history was significant for regular binging and purging in the six months prior to presenting, although at the time of initial diagnosis her eating disorder was restrictive in nature. At the time of the emergency visit, the patient’s weight was 47.3 kilograms, and her body mass index (BMI) was 18.5 kg/m^2^. At triage, the patient reported being unable to have a bowel movement in the week prior to presentation. The patient reported the need to severely “strain” to try and pass stool but in doing so, had experienced a rectal prolapse. The patient was able to digitally correct the prolapse without issue but became nauseated in the moments thereafter and subsequently vomited. The patient informed her mother of the prolapse and they presented to the ER given her general state of feeling unwell. On the way to hospital, the patient also developed a severe headache. Her vital signs at triage showed a heart rate (HR) of 72 beats per minute (bpm), temperature of 35.5 degrees Celcius oral, and lying blood pressure of 94/63 mm HG. The patient reportedly looked unwell and complained of dizziness. Over the course of the next 60 minutes, the patient’s headache worsened, prompting the emergency physician to complete bloodwork and start an IV. Her bloodwork revealed a low potassium level (3.2 mmol/L, lower limit normal 3.5 mmol/L). An electrocardiogram was completed which revealed normal sinus rhythm and no arrhythmia. The patient’s headache was treated with a single dose of IV toradol 15 mg and her potassium was replenished via IV supplementation. No further history or mention was made of her rectal prolapse during the ER visit, and she was subsequently discharged home twelve hours later with parents after reportedly feeling much better.

The patient followed up in the eating disorder outpatient clinic within the week. The events of the emergency visit were reviewed, and the patient reported ongoing issues with rectal prolapse with bowel movements. It was at this point that she revealed that the prolapse had occurred for the first time shortly after beginning treatment in the ED day treatment hospital. This correlated with an increase in her weight given improved nutritional delivery, a decrease in eating disorder symptoms, but also a stooling pattern which occurred on average once weekly. Historically, the patient had disclosed issues relating to her constipation (but not the prolapse) and had been treated with a combination of increased fluid and fiber, as well as docusate sodium 200 mg daily and eventually a trial of PEG 3350 which provided relief. Over the next 2–3 months her constipation (and prolapse) gradually improved. The patient noted increased anxiety at the time of her discharge from intensive ED services, and relayed part of her fear to worries that the constipation and prolapse would recur. As a result of this fear, the patient consciously began restricting and purging in an attempt to limit the amount of nutrition that passed through her gut and bowel. In doing so, her weight dropped and unfortunately she once again became constipated, which ultimately resulted in a recurrence of the prolapse.

The patient was referred to the division of general surgery. Physical examination confirmed the presence of the prolapse (evident with abdominal straining) and the patient was counselled on the need to restart her fiber supplement as well as reinitiate PEG 3350 daily. She was also taught Kegal exercises as a means of strengthening her pelvic floor muscles (thought weakened as a result of her overall malnourished state). Gradually, over the next six months the patient once again became symptom free from her eating disorder and her constipation and prolapse resolved completely. She has remained at a healthy weight over the last twelve months and has not experienced any further recurrences of her prolapse.

## Discussion

Rectal prolapse manifests as protrusion of the full-thickness of the rectal wall through the anal canal and may occur as a result of a variety of risk factors in patients with severe eating disorders (EDs) [9]. It has a bimodal peak and is more commonly seen in extreme ages. In the pediatric population it usually presents before age four, has no sex predilection, and is associated with chronic constipation, cystic fibrosis, and other medical conditions [10]. In contrast, in the adult population the elderly are more vulnerable and peak incidence occurs in the seventh decade [11]. Women are six times as likely as men to have rectal prolapse. A consensus among experts in regards to a theory detailing the exact pathophysiology of rectal prolapse remains elusive. However, it has been proposed that the anatomical basis for rectal prolapse involves pelvic floor muscle weakness allowing the rectum to herniate through [12]. Diastasis of levator ani, dilatation of the anal sphincter and detachment of rectal sacral ligament have also been implicated as exacerbating factors [13,14].

Predisposing risk factors for rectal prolapse typically include previous pelvic surgeries, obstetric trauma, elevated intra-abdominal pressure, advanced age and chronic constipation [15]. It is thought that the descending bowel into the rectum may cause a mechanical blockage that is worsened with persistent straining, pelvic floor muscle incoordination and colonic dysmotility [16,17]. Associated symptoms of rectal prolapse can be particularly worrisome as they include reducible protruding mass with bowel movements, mucous discharge, feeling of incomplete evacuation, rectal bleeding, change in bowel habits and fecal and/or urinary incontinence [11].

The association with EDs and chronic constipation has been well-documented [18,19]. Risk factors for constipation that pertain to young women with eating disorders include female gender [20], low caloric intake, low-fiber diet [21,22] and potential polypharmacy [23]. Slowed colonic transit time is more common in women [20], and this is particularly characteristic of women with eating disorders [24]. Medications and/or organic medical conditions can also contribute to secondary constipation. Eating disorder patients are often prescribed medications that worsen constipation, such as, antipsychotics, antidepressants, and diuretics [25]. Also, they are predisposed to co-morbid conditions, including depression, irritable bowel syndrome, and cognitive impairment. In 2004 Marceau and colleagues illustrated that young patients with chronic psychiatric disorders receiving long-term medications had an increased incidence of rectal prolapse with markedly poor prognosis [26].

Although the relation between chronic constipation and AN has been documented previously, its underlying etiology is not yet clearly understood. Increased defecatory perception thresholds and altered expulsion dynamics has been theorized [24]. Chronic constipation may lead to bloating, abdominal distension, and early satiety leading to pervasive “feelings of being fat” rendering AN remarkably resistant to weight restoration [18]. These symptoms may promote laxative and diuretic abuse resulting in electrolyte abnormalities.

Delayed solid gastric emptying witnessed in AN may worsen chronic constipation and its associated symptoms [18]. It has been proposed that the delay in gastric emptying may be due to starvation [27], protein malnutrition resulting in smooth muscle atrophy of intestinal mucosa [28], gastric dysrhythmias or lack of peristalsis [29], or that rectal distension leads to reflexive inhibition of gastric emptying [30]. Appropriate nutrition improves gastric motility.

In an effort to quantitatively describe constipation severity, numerous studies have examined colonic transit times using radio-opaque marker technique in patients with AN compared with healthy controls [30-32]. Research consistently showed significant delayed intestinal transit times in patients with AN. Subsequent re-testing after re-feeding had occurred revealed normal colonic transit times, resulting in the conclusion that re-feeding and weight restoration ameliorate constipation in affected individuals.

In the case of our patient, a number of indirect risk factors likely predisposed her to developing the prolapse as opposed to any one obvious cause. She had complained previously of constipation and had over the course of the preceding 4–6 weeks been started on a regular progressive feeding plan which resulted in a significant increase in oral intake, along with a stated reduction in ED symptoms. Although not measured, she would almost certainly have continued to experience decreased intestinal transit time in the early course which would have exacerbated her constipation. She acknowledged the need to constantly strain in order to have a bowel movement and this, along with her history of purging would almost certainly have resulted in increased intra-abdominal pressures. Finally, her history of malnourishment likely weakened her pelvic floor muscles (among other muscles) which would have also elevated her risk. Clearly, these risk factors are present in countless patients that present for and progress through intensive treatment. Our patient’s case highlights the need for health care providers to pay particular attention to patient’s medical symptoms as they make their way through treatment and to continue to ask pointed questions even in cases where treatment appears to be progressing without issue.

## Conclusion

Rectal prolapse is a rare occurrence in young women, and appears to be an infrequent secondary GI complication of eating disorders. Witnessed in patients with BN and AN, rectal prolapse likely results as a consequence of a number of different factors some of which are related to malnutrition and others as a consequence of regular purging and constipation. It is possible that greater numbers of women experience this condition, but do not bring it to their health care provider’s attention given their angst and anxiety associated with the problem. This was certainly the case with our own patient. We would recommend that treatment providers include basic screening questions related to rectal prolapse as part of their ongoing review of GI symptoms to ensure that patients feel comfortable in relaying all their concerns at the time of initial assessment but also at regular points throughout treatment. Also, given the fact that prolapse is such a rare occurrence in adolescents, we would suggest that any young women presenting with gastrointestinal complaints, chronic constipation and a history of rectal prolapse be also screened for ED behaviours.

## Consent

Written informed consent was obtained from the patient for publication of this Case report. A copy of the written consent is available for review by the Editor-in-Chief of this journal.

## Competing interests

Both authors declare that they have no competing interests.

## Authors’ contributions

NM completed the literature review and drafted the article. MN conceptualized the design of the paper, wrote the case details, and drafted the article. Both authors have given final approval of the version to be published.
